# P362: Biomedical waste in hospital: the case of maternity Issaka Gazobi and national hospitals of Niamey and Lamorde

**DOI:** 10.1186/2047-2994-2-S1-P362

**Published:** 2013-06-20

**Authors:** H Djibo, M Kamaye, A Baden

**Affiliations:** 1 Department of Public Health, Faculty of Health Sciences, University of Niamey, Niamey, Niger; 2 Control Unit Health Sector against STI / HIV / AIDS / Department of Public Health, Niamey, Niger; 3 National Hospital of Niamey, Niamey, Niger

## Objectives

To study the knowledge, attitudes and practices ofmedical waste management in health in Niger.

## Methods

Preliminary prospective cross-sectional study, conducted over a period of 5 months and 10 days with an observation of the types of medical waste and an analysis of the knowledge and practices of 125 officers from three clinics in Niger. In total there were identified and interviewed in the Maternity Hospital and Issaka Gazobi National Niamey and Lamordé: 40 officials commonly called 'major' service, 61 laborers, 21 operating assistants [BH1], and 3 hygiene and sanitation technicians.

## Results

It showed that the composition of medical waste is almost the same in three institutions. Characteristics of the wasteneedles, syringes, vials of injectable ampoules, gloves, pouches blood, urine bags, tubes, cotton, gauze, plaster, human organ, expired products, pharmaceutical and chemicalwastetotaled49. 60% of the residues services produced by the study. Knowledge of risks associated with medical waste: 92% are aware of the risks associated with mismanagement of medical waste and 64.8% of agent shave more than 5 years in their current position held, 70% of the 'majors' service state medical waste not undergo any treatment in the service and that the three health produce a total23.77tons per week or about 1236.04 tonnes of waste per year.

## Conclusion

The need to strengthen the process of medical waste management in the health facilities.

## Disclosure of interest

None declared

